# Alabama State Dental Association

**Published:** 1886-07

**Authors:** 


### PROCEEDINGS OF SOCIETIES.

### Alabama State Dental Association.

ANNUAL MEETING, 1886.

(Reported for DENTAL REGISTER by "Mrs. M. W. J.")

The Alabama State Dental Association held its annual meeting for the year 1886, in the rooms of the Montgomery Medical Association, April 13th, 14th, 15th, and 16th.

The attendance both of members and visiting dentists was good, and the proceedings both instructive and interesting.

The Association was called to order by the President, Dr. J. C. Wilkerson, of Selma, with the following officers present:

First Vice-President, Dr. H. D. Boyd, Troy.
Second Vice-President, Dr. T. P. Whitley, Wetumpka.
Secretary, Dr. T. M. Allen, Eufaula.
Treasurer, Dr. G. M. Rousseau, Montgomery.

The first paper:

DENTAL CHEMISTRY AND THERAPEUTICS, read by Dr. S. Rambo, Montgomery, was discussed by Drs. Rousseau, Eubanks, and Chisholm.

The attendance was small on the first day, travel on all the railroads leading to Montgomery being delayed by washouts from the unusually high water of all the rivers of Alabama.

Many arrived during the night, including Professor J. H. Coyle, Thomasville, Georgia, Professor of Operative Dentistry in the Baltimore College; Professor Wm. H. Morgan, Vanderbilt University; Dr. J. R. Walker, New Orleans; Dr. H. B. Williams, Mississippi; Messrs. I. C. Morrison and J. D. Herbelin, of the Tennessee Dental Depot. The latter gentlemen made a fine display of dental goods in the ladies ordinary, of the Exchange Hotel.

The morning session was opened with prayer by Professor Wm. H. Morgan.

*Dr. H. D. Boyd*, Troy, read a brief paper on

### COHESIVE FOIL.

This was mainly a report of extensive operations in restoring the contour of the six anterior superior teeth, which had been previously filled six times in fifteen years, each tooth having cost the patient over $100. So much tooth-substance had been lost by the file, by fractures, and by decay, that but little was left but a " tumble-down ruin." The methods employed in preparing the cavities, and using the gold, were minutely detailed. The nearly or quite exposed nerves were delicately covered with oxy-phosphate, and the fillings made with No. 4 cohesive gold, the operator using an ordinary mallet, in his own hand, as preferable to either " machine mallets or well-trained assistants." Space was obtained by wedging, the file being " the poorest of all means, and the most offensive to the patient."

This paper being the report from the Chairman of the Committee on Operative Dentistry, the discussion which followed was not confined to the subject of the paper.

*Prof. Wm. H. Morgan*, Nashville, opened the discussion. He was severe in the condemnation of the practice of cutting deep cervical grooves, as tending to impair vitality. The tubuli being cut across, the outer flange of dentine was devitalized—cut off from the pulp, the source of nutrition, and decay would soon ensue. In case of approximal cavities extending to the cutting edge, he would carry the gold across the cutting edge. With patients under thirty years of age, would always, with such approximal cavities, build across the cutting edge, even if necessary to shorten the tooth and build down again. He considered this the only way to insure a permanent operation. He commended the old Johnson's gold, which hardens under the mallet; also Abbey's gold, and the Globe Foil, originated by David Morgan. Dr. Morgan was severe in condemnation of the practice of annealing their own gold by the individual dentists. That in the manufactory there was one man who made it his special business to handle each sheet separately, having special facilities for making it perfectly uniform, which could not be done in the office. The alcohol in the lamp might be too low, or the wick too high, or the atmosphere of the room impure from burning coal or gas, or the fumes of ammonia, etc.

*Dr. Boyd* thought that every dentist was liable to find times when his gold "would not work," and it became necessary for him to anneal it in the flame of his lamp. If he depended on always using it, just as it came from the depot, he would be "badly left" sometimes. The only essentials were to have the lamp clean, and the alcohol as nearly pure as possible, heating the gold to redness in the clear flame.

*Dr. Morgan* said that when cohesive gold was properly prepared it would not deteriorate. Molecular changes were induced in heating, and by passing it through the flame it was no longer pure though it might be freed from foreign substances on the surface.

*Dr. Walker* seconded Dr. Boyd's objections to the file, which he considered the worst mistake ever made. Teeth that were isolated by the use of the file soon came together again in seeking support, reducing the arch, and bringing flat surfaces in contact, which would soon be attacked by decay. He commended the use of the plastics as taking hold of the tooth and having a chemical action preservative of the tooth, the action of the gold being purely mechanical. Would always fill very large cavities with the cements, lining the walls perfectly, and cutting an anchorage in the hardened cement for a gold surface, using platinum-gold for a cutting edge. For visible cavities in the anterior teeth, use the cements most nearly approaching tooth color, renewing when necessary. In some mouths the cement fillings last for years—in others they dissolve more quickly, but would rather rebuild cement every year than build out a glittering gold mass in the front teeth—though formerly for many years he had taken great pride in his contour work. Teeth which would continue to decay under gold could be saved with phosphates.

*Dr. W. D. Dunlap*, Selma, would build across the cutting edge, as described by Dr. Morgan. If but one approximal cavity, reaching quite or nearly to the corner, would build across in shape of the letter L, or the reverse. Would never leave a thin cutting edge. The kind of gold used or the question of annealing should be left to each operator.

*Dr. Walker* urged the importance of impressing upon patients the necessity of proper care of the teeth, and of keeping up the character of tooth-substance, by such diet and other means as would supply the necessary elements. Teeth will of course decay again no matter how well the work of filling may be done if the teeth are not properly cared for. We must preach as well as practice.

*Dr. Boyd* considered gold the best material in all cases, and failures the fault of the operator.

*Dr. T. M. Allen*, Eufaula, took issue on both points, everything depending on the character of the tooth to be operated on.

*Dr. W. H. Morgan* said that he agreed with Dr. Walker as to the value of the cements. They would key to the walls of the cavity, making absolute contact at every point, working its way even into the mouths of the tubuli.

*Dr. Morgan* claimed to be the first "on record" in the method of filling with cement and finishing with gold—the one who "gave it away," though, Dr. Redman, of Louisville, used it earlier. He objected to retaining points, except in the cementum, which has a living wall beyond, another source of vitality. Cutting through tubuli cut off the source of life in dentine.

*Dr. Boyd* asked why, that being the case, a devitalized tooth lasts longer than a live tooth?

*Dr. Morgan* denied that such was the case, a devitalized tooth having no power of resistance—though a tooth might continue to exist for a while without a pulp, there was a constant retrograde movement—a crumbling away—going back to dust.

*Prof. Coyle* while admitting the truth of the statement, that the microscopical arrangement of the tubuli being cut, the periphery of the dentine was devitalized, being cut off from the source of nutrition, the pulp—yet the question was, what actual effect does it produce?

*Dr. Morgan* said, if the patient lived long enough, that portion of dentine would decay.

*Prof. Coyle* said that while theoretically a devitalized tooth was subject to decay, practically, it as a rule did not decay, which was the paramount consideration.

*Dr. Morgan* said that though the tooth might not decay during a lifetime, yet it would if sufficient time was granted. The theory held good, and that he was the first on record for the theory, as also for the theory of the vitality of enamel.

*Dr. Walker* said that, as a rule, devitalized teeth had great powers of resistence. The function of nutrition was two-fold; the supply of pabulum, and the removal of debris. If proper elements of tooth-structure were not sufficient to a living tooth, the debris would continue to be removed by the function of nutrition, and the waste material not being replaced, the live tooth would go to pieces, where a devitalized tooth, with the albumen well carbolized, would remain good for a lifetime, with the same environment.

*Dr. Morgan* said that he denied the premises, though he admitted the results. A paucity of tooth-building elements was not possible. There was not a place on the face of the earth where ample supplies could not be obtained, even the poorest article of food, as rice for instance, furnishing more of bone and tooth elements than nature calls for to rebuild the skeleton in seven years.

*Dr. Walker* said that if the supply of lime-salts could be limited to the exact quantity found in the skeleton, why could not the supplies of other elements be limited in the same proportion? Why should we take in 100 parts of oxygen for every four parts utilized? Nature requires abundant supplies to allow for waste and elimination.

*Dr. E. S. Chisholm*, Tuscaloosa, returning to the paper of Dr. Boyd, said that all exposed dentine should be covered with gold, and the teeth made to touch at a rounded point near the cutting edge. That patients should be taught the paramount importance of strict cleanliness, and the prevention of any accumulation of food. They should be taught that fillings would not preserve teeth that were not cared for properly. He also inculcated the use of cement for saving poor teeth.

*Dr. Morgan* said that though the cement would wash out badly in some mouths, it would often adhere to the walls of the cavity and preserve from further decay, even where badly washed out in the centre.

*Dr. R. U. DuBois* said this was also the case with gutta-percha fillings, which would wear in a saucer shape, and still protect the tooth from decay.

By request Dr. Coyle gave the history of chemical operations in sponge grafting, in the Baltimore College Infirmary, describing in detail the methods as taught by Dr. Wm. H. Atkinson.

*Dr. Morgan* described the three processes of Nature, in disposing of foreign bodies: by encysting, as in case of a bullet; by suppuration and gradual ejection through contraction, as of a splinter; or by absorption, as with the sponge-graft, animal sutures, etc.

*Dr. J. R. Walker* described some cases of granulation and restoration of gum-tissue, where teeth had been denuded by pyorrhea alveolaris. The cases were still under treatment, fully two-thirds of the tissue having been already restored, with firm attachment, where the periosteum was healthy. One of the patients was a man sixty years of age.

*Dr. Morgan* considered this an important step in general progress.

*Dr. W. D. Dunlap*, Chairman of the Committee on Physiology and Etiology, read a paper entitled:

### THE TEETH AS DERMAL APPENDAGES.

He described the human body as a strange and complicated piece of mechanism, hinged and double hinged—ball and socketed together, with longitudinal and horizontal, and striated layers, piled up one on another, the whole covered with a texture modified to meet the demands of the different parts. He described the mouth as a small but complete mill, requiring skilled labor to keep it in order, and a skilled millwright to make repairs. He described the functions of the skin, as an organ of touch; as a defence of the nerves and blood-vessels; as an organ of absorption and secretion, the mucus membrane being but a continuation of the external skin, for the similar protection of internal parts, and the teeth dermal appendages developed from the mucus membrane along the edges of the maxillary arches. He traced the origin, growth, and development of the teeth through the successive stages.

The skin being the instrument through which the teeth are formed and nourished, whatever impairs the functions of the skin is also deleterious to the teeth, as seen in the effects of various eruptive diseases, the teeth, in their formative stage, sharing with the skin in the lack of nourishment. A scrofulous subject has chalky and dissolving teeth ; a syphilitic taint is marked upon the teeth, and so of other diseases, these relations meriting more careful investigation than they have yet received.

*Dr. Morgan* opened the discussion of this interesting paper by describing some very marked cases he had seen of the specific marks of syphilitic poison on the teeth; marked by a great deficiency of earthy matter in the dentine—the pulp chambers and nerve canals abnormally large, the foramen broad open like a funnel, etc.

In a scrofulous diathesis the teeth are pearly in appearance--a beautiful type of teeth to the ignorant eye, but which succumb readily after the development of pulmonary symptoms. In the other form, where the glands are involved, the enamel is softened and the dentine breaks down readily, the gums being inflamed with a fetid discharge. These are the usual characteristics of the teeth of the negro race. When "fed about the house" they rarely have sound teeth at maturity ; this is especially true of mulattoes.

Dr. Morgan also said that he was satisfied that the teeth do suffer similarly with other dermoid structures and appendages, but the injury is more serious as imperfect crystallization of the enamel is never obliterated. An abscess of the deciduous tooth may through inflammation of the mucous membrane injure and mark the permanent tooth. The vitality of a bicuspid may be destroyed by a diseased deciduous molar.

*Dr. E. S. Chisholm* said that he had often traced affections of the teeth clearly to severe cutaneous diseases, all of the teeth which were at the same stage being similarly marked.

*Dr. Walker* thought that a scrofulous diathesis was often due to excessive use of hog products. That an entire change of diet in this respect would often produce a marked change in the system. That this was intensified in certain Northern States, where the percentage of deaths from scrofulous and pulmonnary troubles was very high, by living in close overheated rooms a large part of the year. Southerners are also large pork consumers, but live much in the open air. The corn bread and and hominy eaten with bacon, etc., in the Southern States, further neutralizes its bad effects, and lowers the death rate from scrofulous affections.

*Dr. T. M. Allen*, Eufaula, read a paper on

MECHANICAL DENTISTRY.

This was followed by a discussion of the comparative merits of the various materials used for plate-work. Nothing new was elicited. Dr. Walker gave his method of seasoning celluloid, which he uses almost exclusively, having discarded rubber many years ago. By seasoning a celluloid plate over a low heat, after it is pressed and the flask closed, for a length of time proportionate to the time employed in pressing, the camphor is evaporated, the material becomes hard and fine, retaining its color and shape well, and fitting it for permanent work, excelled only by continuous gum, and equalling even that in capacity for artistic finish and adaptation, with the advantage of being much lighter, and also very much cheaper. Proficient, skilled, conscientious workers in celluloid find it a clean, substantial, durable, artistic substitute for all other materials for either partial or full sets of teeth.

*Dr. Eubanks* agreed with Dr. Walker as to the value of celluloid for permanent work. He uses the New Mode Heater. Plates that he made five years ago still retain their perfect fit and color.

*Dr. Morgan* commended the use of vulcanizable gold for lining rubber plates, as obviating the troubles caused by contact of the rubber with the mucous membrane, and offering all the advantages of a gold plate as far as the upper surface of the plate is concerned. Attributes the trouble to roughly made, badly fitting plates, and considers rubber a blessing to thousands of people who would otherwise have " gummed it" to the end of life. Did not think that celluloid would answer for partial pieces or permanent plates—would as a rule deteriorate within a year. Admitted its great capacity for the display of artistic skill, but thought the man capable of really artistic work would not put it on a perishable material like celluloid at celluloid prices. He did not think the non-conducting properties of rubber had anything to do with the troubles complained of, as heat-elimination was not a function of the mucous membrane. If there was abnormal heat under the plate it was the result of inflammation, due to irritation from roughness or bad fit of the plate. Black rubber, though equally a non-conductor, was admitted by all to be more compatible with the membranes. The subject was discussed at some length without reaching any satisfactory conclusion.

By request Prof. Coyle spoke of bridgework, commending it for short pieces attached to sound teeth. Where properly made and applied under proper conditions nothing else would so exactly meet the requirements of certain cases.

He did not approve of very long pieces, nor of attachments to devitalized teeth, or where the peridentium had once been a seat of inflammation. He had worn a piece himself a year and a half, and was scarcely conscious of its presence—considered it preferable to partial plates, which always had more or less lateral motion injurious to the adjacent teeth.

The subject of the treatment of pulpless teeth was discussed at length.

*Prof. Coyle* said this subject had furnished more food for discussion both in Associations and journals than any other one topic before the profession. He wished to make clear the distinction between *pulpless* and *dead* teeth. Pulpless teeth were not necessarily dead teeth. Where the attachments to the alveolus were intact there was still a means of nutrition. When the pulp-tissue was removed, and septic conditions insured, the root canals and pulp chambers filled, the tooth could be restored to years of health and usefulness, though the investing membrane—the only connecting link between the tooth and the vital economy was, of course, still liable to disease. He would remove the larger part of the dentine, leaving only the cementum, which was nourished by the peridental membrane and not influenced by the absence of pulps. Would not use carbolic acid in early dressings as it would block up the mouths of the tubuli, and prevent carbolization of the contents. Would dress with a mixture of

Eucalyptus oil, 1 oz.
Iodoform, 1 drachm.

and fill with an orange wood peg trimmed to fit the canal, dipping it in a preparation of equal parts of gum camphor, chloral hydrate, carbolic acid crystals, if septic conditions had existed. If trouble ensues, open through the alveolus, burr off the end of the root, if necrosed; and treat with iodine, either alone or combined with creasote, on a tent of floss silk. The subject was discussed by Drs. Dunlap, Rousseau, Eubanks, Chapman, T. M. Allen, Coyle, Walker, and Morgan.

*Dr. Rousseau* treats with iodoform and fills with gutta-percha, pushed down with a heated Donaldson bristle point. Did not see how the wooden peg could be trimmed to fit a root canal, and had known of one being so thoroughly driven down as to be driven through, and removed from under the tongue! He would treat and heal everything up before filling.

*Dr. J. R. Walker* gave his method of obtaining a model of the root canal, by which to shape his wooden root filling. He wraps a few fibres of cotton on a broach in which he intermingles little pieces of parafine. Careful removal gives quite an exact model. He fills with chlor-percha, driving the peg in to force the plastic into all the interstices. For small or crooked canals, the chlor-percha alone would be found sufficient.

*Dr. Morgan* emphasized the distinction between dead teeth and pulpless teeth; also between "treating a tooth" and treating the membrane, and the use of the words antiseptic and disinfectant as implying the same thing. A disinfectant destroys the products of decomposition; antiseptics prevent their production. He considered immediate filling bad practice, the tubuli being full of semi-fluid matter, there being as much soft material in the tubuli as in the entire pulp, and equally liable to decomposition, putrefaction, and discoloration. Would treat with carbolic acid. If the albumen is carbolated there can be no decomposition. Did not think root canals could be successfully filled with solid materials alone without much cutting out of the canal. He enlarged with a cone-shaped drill, and filled with a gold screw, cut to correspond with the drill.

*Dr. Geo. Eubank* said that it had fallen to him to extract a great many dead teeth, the roots of which he usually found filled with the plastics—rarely with gold. He filled roots with creasote and cotton.

*Dr. T. G. Robertson* filled roots with fluid phosphate, pumped in with a broach wrapped with cotton, which acts as a piston, forcing out the air and driving the fluid to the end of the root. Subsequent extractions had proved this operation to be very perfect.

The subject of replanting teeth was discussed. Prof. Coyle said that he had had marked success. With a separating file he cut a groove lengthwise of the root, for the escape of serum from the socket, without which precaution there was liability of subsequent soreness. Used tincture of aconite to prevent tendency to inflammation. A tooth should never be replanted with an abscess in formative stage. The parts must be perfectly sound and healthy. If the alveolus was necrosed would not replant. Would not extract pulpless teeth except as the last resort. A replanted tooth would often become the firmest in the mouth.

At the third morning session, Prof. J. H. Coyle, Thomasville, Ga., of the Baltimore College, and Dr. and Mrs. J. R. Walker, New Orleans, were elected honorary members of the Alabama State Dental Association.

*Dr. Geo. Eubank* read a paper on

### DENTAL EDUCATION.

He spoke of the question of "recognition" now agitated in the profession, and said that when through higher education we were able more thoroughly to respect each other, the public would respect us. We must hold out our hands to the weak brethren and strive to lift them up. Through education alone the future was within our control.

He argued strongly against the system allowed by the Dental law of Alabama, of granting temporary licenses to students. It was done on the pretext of giving them a chance of making money to go back to college, but as a rule, if they got to making money they would be self-satisfied, and seek a renewal of the license instead of going back to college He considered it an imposition upon the public. A young man who had spent a few months nominally under a preceptor, but really watching a vulcanizer or polishing plates, armed with such a license could scour the country, extracting teeth and putting in rubber plates, under the title of doctor! It was no wonder that the physician objected to having that honored title so dragged down, with no distinction made between the ignorant charlatan and the skilled, educated scientific dentist. With the law properly administered, and the Dental Examining Boards composed of competent men, doing their duty fearlessly, rejecting all who were found incompetent, the public would no longer be imposed upon as in the past. The past was irretrievable, the future is in our hands.

*Dr. Walker* congratulated the Society on having listened to a sound paper. In the past men who had made failures in everything had "taken up dentistry," with the idea that it was an easy method to make money. He objected to the founding of so many dental colleges and dental departments. It was a political scheme for the benefit of men ambitious of holding office. Our influence should be thrown in favor of the colleges which had been tried and found trustworthy.

*Dr. W. D. Dunlap* said that the cry for "recognition" was a tacit admission of inferiority on our own part. He had never found any lack of recognition where there was a call for it. Physicians were always ready to consult with dentists of good standing when the field of operation was mutual. He did not like the term. The negroes too were calling for recognition. Dentistry is our profession, and he was satisfied with it, ignoring the medical profession.

*Dr. Walker* said that we were as much members of the medical profession as the oculist or the aurist, requiring fully as much knowledge of the science of medicine.

*Dr. Morgan* said that the after history of the young men who had been the most distinguished at college did not as a rule show practical success. A medalist was seldom heard of after he left college, rarely becoming a leading man in his profession. There had been eminent men in dentistry long before there were any dental colleges. What had been done in the past could be done in the future. He had never seriously felt the need of Latin or Greek, and did not see any advantage in covering up very simple things with very long names and then stopping to explain them. If they were used in the books, so much the worse for the books. That some of the most beautiful fillings—very difficult operations—had been made by a three months student.

*Dr. Eubanks* said that did not qualify him to go out and practice dentistry, or imply that he had the ability to save the natural organs, or that he would make a success as a practical operative dentist.

*Dr. DuBois* did not think that first year students should be licensed to go out into the country and practice on the people; they should work with older practitioners.

*Dr. Morgan* said that the phenomenal growth of dentistry as a profession was due to the fact that it had not been burdened with musty authorities or obliged to endorse what was thought five hundred years ago. Each man had to investigate for himself. Much that was taught in the colleges was of no practical use to the practical dentist. Valuable time was spent in learning things for which the student would never have any use. He was in favor of allowing young men to practice wherever they could find practice. It was as correct for them to practice on a community as in an infirmary; as well to get something for his work, as to get nothing. A failure in the one case, was no worse than a failure in the other, they were our fellow-men in either case.

*Prof. Coyle* being called upon said that he responded with hesitancy as he had not heard Dr. Eubank's paper, but he would reply to Dr. Morgan, whose remarks he had listened to with interest. He had uttered some profound truths. Not all education educates, much that passes for such does not aid in fitting a man for his avocation. Education cannot put into a man what is not there; it can only develop the native material of the mind; only bring out what is in him—like the germ in the soil, waiting for light, heat, and moisture that it may put on its robes of beauty. A man who is able to appreciate and grasp the idea that he is to operate on living tissues must be educated before he can understand its functions and physiology. He must understand something of general pathology before he can treat oral lesions; anatomy and the microscopical development of the teeth to deal successfully with pulpless teeth; but he must also acquire that mechanical handicraft which is indispensable to the practical operative dentist.

The colleges have done good work for the profession, but the curriculum and the standard for graduation, and especially the standard of admission require raising.

*Dr. Morgan* said there were many men who could treat and save decayed teeth, and who could treat Rigg's disease—but who is there that understands the pathology of caries—the pathology of Pyorrhea Alveolaris?

*Prof. Coyle* replied that when we speak of understanding we mean within the present limits of investigation. Our future therapeutics will be adapted to future revealments.

*Dr. T. P. Whitley*, Wetumpka, read a paper entitled:

### DENTAL EDUCATION AND LITERATURE.

The discussion of this paper reopened the questions previously discussed.

*Dr. E. T. Chisholm* said that the present great need of the profession was text-books better adapted to the wants of students, and commensurate with the present standing of the profession and its recent rapid growth—books which start further back in elementary principles, and books up to the present scientific standard.

The subject of temporary licenses to students was brought up again. Dr. Walker thought they should acquire their first experience in the infirmary of a dental college, where their work would be carefully done, under the supervision of instructors, and done for people who were willing to take the risks on condition of not paying for it. While gaining this experience they would perhaps save teeth that would otherwise be lost. He would give the young men every opportunity, but not at the expense of those who were able and willing to pay for what they supposed to be good operations. Outside experience was a damage both to the student and to the profession, and should not be encouraged. The object of dental laws was to protect the people from just such practices, and the temporary license was an evasion of the law.

*Dr. S. G. Robertson* thought that students who had no opportunity for practice during vacation would never make dentists. It was the duty of those already in practice to help them along, and give them opportunities. He, for one, was willing to open his office to them.

*Dr. W. W. Evans* thought that young men fresh from college were often much better prepared than older men, and he could not see that it would be any imposition upon a community if the community was willing; it was a matter of free choice.

*Dr. Walker* said they were blinded by the license granted.

*Dr. Dunlap* said he was not willing to allow inexperienced young men to go out under the sanction of the Dental Board of the State. If colleges and preceptors were not doing their duty he wanted them kept up to the standard. Some men can acquire more theoretical knowledge in three months than others in three years, but that does not imply practical competency. Even a shoemaker cannot become competent in three months. And yet we see dentists, so called, turned out in four or five months!

*Dr. Eubank* said he knew of one of the latter class whose first patient was a young lady of fourteen summers, for whom he had extracted the canines because they were outside the arch.

*Dr. Coyle* said a first year student was only on the threshold of the knowledge and experience necessary for general practice. If it was necessary for him to make money, he could go back to the dental infirmary where he would be charged nothing for bringing in his paying patients, and where his work would be under proper supervision. In that way the law would be obeyed, the public would be protected, and no harm done to anyone.

*Dr. Rousseau* said that license was granted only at the annual meeting of the Board, and only on satisfactory evidence of graduation or of competency; but that temporary licenses were granted during the interregnum. Such licenses expire on the day of the annual meeting, and but one temporary license is granted to any man. A permanent license had to be recorded in the Probate Court at the place of his residence, and renewed in case of change of residence.

*Dr. R. U. DuBois*, Greensboro, read a paper on

CHEMISTRY

which was not discussed.

The night session of the third day, was devoted to

INCIDENTS OF OFFICE PRACTICE.

*Dr. Dunlap* related a case, in which after extraction of a lateral incisor, the patient had been instructed to treat the abscess with aromatic sulphuric acid, injected with a hypodermic syringe, which he carried in his vest pocket and used *ad libitum.* After keeping this treatment up for three months, the fistula continuing to enlarge instead of healing, he consulted Dr. Dunlap. Examination showed a piece of necrosed bone back of the original line of the tooth-socket. The bone was loose and could be moved about. It was removed with surgeon's forceps, and proved to be the inferior turbinated bone.

*Dr. J. R. Walker* described a case of necrosis, which he had cured without the loss of a tooth. The right superior bicuspid was dead, with an active abscess opening both outside and inside, proving to be a very obstinate case. The tooth was put in sanitary condition and filled, but there was still necrosed bone above and behind the root, which he treated through the alveolus, burring out all the necrosed bone and restoring the parts to health, and saving all the teeth. He uses permanganate of potash as a preliminary wash, the too early use of carbolic acid was liable to arouse active conditions.

*Dr. S. Rambo* said that Darby's Prophylactic Fluid owed its efficacy partly to permangnate.

*Dr. Geo. Eubank* gave the details of severe injuries received by the caving of a mine and the falling of a heavy mass of coal on the head of a miner. The superior maxillary was fractured behind the wisdom tooth, and also near the median line. The lower jaw was broken in three places. In pressing the superior maxillary into place the lower jaw sprang out of its socket and was very difficult to replace. Two bicuspids and the first molar were knocked out. These were put back and ligated, the pieces of the jaw were all brought into position, the teeth knuckling together nicely. The pieces of the upper jaw were perfectly united in less than a month. There was no abscess from the replanted teeth, all the fractures united nicely, the mouth opened almost normally with the articulation almost as perfect as ever. Dr. Dunlap reported a somewhat similar case.

*Dr. E. S. Chisholm* gave the details of some interesting operations in correcting projecting superior maxillaries by extracting the six anterior teeth, laying back the gums and trimming off the alveolus with wedge forceps to the posterior line of the sockets, making a plate with the front teeth antagonising properly with the lower teeth before extraction, and inserting immediately; the extraction of the teeth and trimming the alveolus not taking more than ten minutes. The permanent plate was made a few months later.

*Dr. J. R. Walker* preferred to avoid a plate if possible. He reported a case of very projecting superior maxillary, which he had corrected by sacrificing only the very defective first molars. The anterior teeth were sound, but very irregular, and the jaw projecting fully one-half inch beyond the lower. He had brought them with him, and ligated to a retaining plate applied to the lingual surfaces. Those being in anchorage within the mouth he had applied to the labial surfaces a brace or bar of hickory wood, which projected from the corners of the mouth, extending past the sides of the head. To the extremities he attached an elastic band which passed around the back of the head, supported by a cushion. The constant pressure had caused contraction of the arch, and the teeth had been brought into normal articulation and regular arch.

*Dr. Geo. Eubank* reported a case of anchylosis.

The patient, a boy of nine years of age, had an attack of Typhus fever, treated with calomel with severe salivation, sloughing being very extensive with the loss of three teeth on one side and four on the other. After several days of delirium, the jaws were found to be firmly set, and had been absolutely immovable for ten months, when Dr. Eubank first saw him. He had been fed through the space left by the missing teeth. Under the influence of ether, efforts were made to pry open the jaws, but it was found that they would break, but not yield the one hundreth part of an inch. Dr. Eubank considered that in case of regurgitation of food the boy's life would be endangered through suffocation.

*Dr. T. M. Allen* reported a similar case, but in which he had been able to open the jaws about an inch, by the use of strong strings and wedges. Dr. Walker exhibited the models of some interesting regulating cases. In one case, where the lower jaw was naturally quite protruding the deformity had been increased by malpractice, three sound teeth having been extracted in the effort to bring the superior canines into line, the result being such a contraction of the arch that the upper front teeth fell entirely inside of the lower, the labial cusps of the superior molars falling on the lingual cusps of the inferior, the patient being unable to either eat or speak with any comfort.

Dr. Walker had expanded the arch sufficiently to replace the three missing teeth, and by drawing in the lower front teeth had produced a very fair articulation all around.

He exhibited the casts of a number of other cases, with the fixtures used in regulating—modifications of the coffin plate, etc.

This feature of the history was much appreciated as being a method of practical information—and as opening up a most instructive field.

*Dr. Boyd* showed a small appliance of zinc, acting as an inclined plane to lift out a tooth from inside of the arch, biting upon it gently lifting the tooth forward.

*Dr. Walker* said that that simple fixture, so easily applied, was sufficient to pay him for his trip from New Orleans, if he had not already been amply repaid.

*Dr. Dunlap* said it was so simple and so feasible that it looked as if everybody ought to have known it all the time, only they didn't think of it.

*Dr. Walker* thought that the more attention was given to the teeth, the more their importance would be appreciated—compared to it, filling teeth and making plates was mere patchwork. By presistent correction of deformities and irregularities the tendency in that direction could be overcome.

Local anæsthesia was discussed—cocaine was found generally unreliable; cannabis indica very valuable.

*Dr. Coyle* preferred ether spray. Equal parts of aconite root and chloroform often gave fine results. Hypodermic injections of cocaine, if made with a thorough knowledge of the anatomy of the parts, was effectual but almost as painful in itself as the extraction of the tooth.

At the final session, after passing the usual votes of thanks to the railroads, hotels, the press, etc., including "Mrs. M. W. J." for report of proceedings, the election of officers was held, with the following result:

President, R. U. DuBois, Greensboro.
First Vice-President, T. P. Whitby, Wetumpka.
Second Vice-President, W. W. Evans, Union Springs.
Secretary, T. M. Allen (re-elected), Eufaula.
Treasurer, G. M. Rousseau (re-elected), Montgomery.
Executive Committee, E. S. Chisholm, Tuscaloosa; G. M. Rousseau, Montgomery; Geo. Eubank, Birmingham.
Place of next meeting, Tuscaloosa.
Time of meeting, Second Tuesday of April, 1887.

The sight, odor, or thought of agreeable and nutritious articles of food *will often stimulate the salivary fluid when* mastication and deglutition are quiescent.

